# Quantitative analysis of respiration-induced motion of each liver segment with helical computed tomography and 4-dimensional computed tomography

**DOI:** 10.1186/s13014-018-1007-0

**Published:** 2018-04-02

**Authors:** Yu-Lun Tsai, Ching-Jung Wu, Suzun Shaw, Pei-Chieh Yu, Hsin-Hua Nien, Louis Tak Lui

**Affiliations:** 1 Department of Radiation Oncology, Cathay General Hospital, Taipei, Taiwan; 2 Department of Radiation Oncology, National Defense Medical Center, Taipei, Taiwan; 3 Department of Biomedical Engineering, I-Shou University, Kaohsiung, Taiwan

**Keywords:** Liver segment, Respiration-induced motion, 4-dimensional computed tomography, Deformable image registration

## Abstract

**Background:**

To analyze the respiratory-induced motion of each liver segment using helical computed tomography (helical CT) and 4-dimensional computed tomography (4DCT), and to establish the individual segment expansion margin of internal target volume (ITV) to facilitate target delineation of tumors in different liver segments.

**Methods:**

Twenty patients who received radiotherapy with CT-simulation scanning of the whole liver in both helical CT and 10-phase-gated 4DCT were investigated, including 2 patients with esophagus cancer, 4 with lung cancer, 10 with breast cancer, 2 with liver cancer, 1 with thymoma, and 1 with gastric diffuse large B-cell lymphoma (DLBCL). For each patient, 9 representative points were drawn on the helical CT images of liver segments 1, 2, 3, 4a, 4b, 5, 6, 7, and 8, respectively, and adaptively deformed to 2 phases of the 4DCT images at the end of inspiration (phase 0 CT) and expiration (phase 50 CT) in the treatment planning system. Using the amplitude of each point between phase 0 CT and phase 50 CT, we established quantitative data for the respiration-induced motion of each liver segment in 3-dimensional directions. Moreover, using the amplitude between the original helical CT and both 4DCT images, we rendered the individual segment expansion margin of ITV for hepatic target delineation to cover more than 95% of each tumor.

**Results:**

The average amplitude (mean ± standard deviation) was 0.6 ± 3.0 mm in the left-right (LR) direction, 2.3 ± 2.4 mm in the anterior-posterior (AP) direction, and 5.7 ± 3.4 mm in the superior-inferior (SI) direction, respectively. All of the segments moved posteriorly and superiorly during expiration. Segment 7 had the largest amplitude in the SI direction, at 8.6 ± 3.4 mm. Otherwise, the segments over the lateral side, including segments 2, 3, 6, and 7, had greater excursion in the SI direction compared to the medial segments. To cover more than 95% of each tumor, the required expansion margin of ITV in the LR, AP, and SI directions were at least 2.5 mm, 2.5 mm, and 5.0 mm on average, respectively, with variations between different segments.

**Conclusions:**

The greatest excursion occurred in liver segment 7, followed by the segments over the lateral side in the SI direction. The individual segment expansion margin of ITV is required to delineate targets for each segment and direction.

## Background

Radiotherapy is an increasingly important modality for treating liver tumors, whether they are primary hepatocellular carcinomas or liver metastases [1–4]. However, respiration causes significant motion of the liver [5]. Variation in the amplitude of motion of more than 5 mm can lead to significant changes in dose distribution [6]. The uncertainty in doses will put normal liver tissue at risk of radiation-induced damage. Patients with radiation-induced liver disease (RILD) also receive a mean dose to the liver that is significantly over their hepatic radiation tolerance and a significantly higher normal tissue complication probability (NTCP) compared to patients without RILD [7, 8]. Understanding how the liver moves during respiration can improve tumor targeting and avoid unnecessary irradiation of normal liver tissue. This is important in treating not only liver tumors but also pancreatic cancers, since the liver is close to the irradiating field for the pancreas. Radiotherapy can be administered successfully with the patient free-breathing by using appropriate asymmetrical expansion [9, 10].

In managing respiration-induced organ motion, a generic but large planning target margin has to be applied when patient treatment plan design based on a single pre-treatment CT scan is used to guide radiation treatment. Planning target margins can be significantly reduced using multiple or 4D image feedback management. One of the most effective methods in multiple or 4D image feedback management of radiotherapy is the adaptive control methodology [11]. Deformable image registration (DIR) is the key component of the image-guided adaptive strategy in radiotherapy [12]. Nowadays, DIR is widely used not only for contouring but also for re-planning, dose mapping, and dose evaluation in many kinds of cancers throughout the body [13, 14]. Several studies have focused on measuring respiration-induced organ motion using DIR [15–17]. Ten more DIR algorithms are available and have been evaluated as registration strategies [18, 19]. SmartAdapt® (Varian Medical Systems, USA) is a DIR tool available in the Varian’s treatment planning system with sufficient accuracy. In a study of validation for clinical application, it showed that only 8% of the DIR-generated structures required major modifications by using SmartAdapt®. Furthermore, 12% to 44% of structures were more accurate than the radiation oncologist re-contoured structures, could be accepted without modifications, or required at most minor modifications upon retrospective review by the radiation oncologist [20].

The consensus on the magnitude of the amplitude of respiration-induced liver motion is not well established [5, 15–17, 21–27]. Furthermore, no clinical studies have assessed the differences between different liver segments. This article explores this issue and provides segment-specific suggestions about ITV expansion to facilitate target delineation.

## Methods

### Patients

Twenty eligible patients who received radiotherapy with CT-simulation scanning were investigated, including 9 males and 11 females whose age ranged from 44 to 85 years old, with a mean age of 58. Patients with all kinds of cancer were eligible. Patients with extensive disease that may immobilize the liver were excluded, such as a tumor adhering to the liver or hepatocellular carcinoma with extra-hepatic extension or ascites. The 20 eligible patients included 2 with esophagus cancer as their primary cancer, 4 with lung cancer, 10 with breast cancer, 2 with liver cancer, 1 with thymoma, and 1 with gastric DLBCL. Regarding the CT-simulation scanning, both helical CT and 4DCT images were acquired at the time of CT simulation. Whole livers and bilateral lungs needed be scanned with both helical CT and 4DCT to be analyzed. The patient characteristics are listed in Table 1.

**Table 1:** Patient characteristics and maximum phase errors of 4DCT

Number	Age	Gender	Cancer type	Max phase error of 4DCT (%)	
				Phase 0 CT	Phase 50 CT
1	80	M	esophagus	9	8
2	54	M	lung	49	7
3	47	F	breast	5	4
4	57	F	breast	7	7
5	70	M	liver	6	5
6	61	F	breast	16	7
7	45	M	lung	14	8
8	52	M	lung	7	7
9	66	F	thymoma	8	7
10	52	F	breast	8	8
11	45	F	breast	7	5
12	52	F	breast	27	8
13	85	M	lung	9	6
14	57	M	esophagus	22	6
15	68	M	liver	9	7
16	63	F	breast	6	6
17	58	M	stomach DLBCL	9	8
18	44	F	breast	27	7
19	64	F	breast	13	12
20	47	F	breast	8	7
mean	58			13	7

Phase 0 CT indicates end-inspiration; phase 50 CT, end-expiration

*Abbreviations: 4DCT* 4-dimensional computed tomography, *M* male, *F* female, *DLBCL* diffuse large B-cell lymphoma

### CT image

Patients were set up in the supine position and immobilized with a vacuum bag cushion system with their arms over their head. Respiratory pattern was neither mentioned nor coached, so that the patients breathed freely with no alteration. Both helical CT and 4DCT scans were made using the Discovery CT590 RT sixteen-slice scanner (GE Healthcare, UK), with a slice thickness of 2.5 mm and a diameter of field of view of 50 cm. The in-field resolution was 1.0 mm. The helical mode was used for helical CT, and the axial cine mode was used for 4DCT. To further describe 4DCT, a mounted detector with infrared ray tracked an external tracking reflector attached to the epigastric area where respiration caused the largest displacement on the surface. The breath patterns of the patients were monitored and recorded with the Real-time Position Management (RPM) Respiratory Gating System (Varian Medical Systems, USA). After scanning, a 10-phase-gated 4DCT image from 0% to 90% distributed over the whole respiratory cycle was constructed from the raw 4DCT data using the Advantage 4D software (GE Healthcare, UK), in which 0% (phase 0 CT) and 50% (phase 50 CT) indicated end-inspiration and end-expiration, respectively. The maximum phase error of 4DCT which is the maximal variability of breath patterns at the specific phase over all breathing cycles is listed in Table 1.

### Procedure

**Fig. 1:**
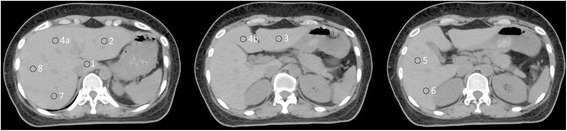
The relative positions of 9 representative points in the liver segments 1, 2, 3, 4a, 4b, 5, 6, 7, and 8, according to the Couinaud classification of segmental liver anatomy

For each patient, 9 representative points were initially drawn on the helical CT images of liver segments 1, 2, 3, 4a, 4b, 5, 6, 7, and 8, respectively, with normal structure boundaries according to the Couinaud classification of segmental liver anatomy (Fig. 1). The Couinaud classification is stated as below:

1. The Couinaud classification divides the liver into 8 functional segments.
2. The portal vein divides the liver into upper (2, 4a, 8, 7) and lower (3, 4b, 5, 6) segments.
3. The middle hepatic vein divides the liver into left and right lobes. The left hepatic vein divides the left lobe into lateral (2, 3) and medial (4a, 4b) segments. The right hepatic vein divides the right lobe into anterior (5, 8) and posterior (6, 7) segments. The caudate lobe (1) has hepatic veins that often drain directly into the inferior vena cava.
4. A line drawn from the middle of the gallbladder fossa to the inferior vena cava roughly divides the liver into left and right lobes. The falciform ligament roughly divides the left lobe into lateral and medial segments (the left hepatic vein is usually located slightly to the left of the falciform ligament).

**Fig. 2:**
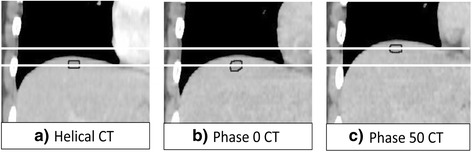
An example of adaptive deformation from (**a**) helical CT to (**b**) phase 0 CT and (**c**) phase 50 CT. The amplitude of liver motion in the SI direction is shown as the distance between the two white lines

Each point was 1 cm in diameter on the axial view along 3 continuous slices. The coordinates of the centroid within the middle slice represented the location of the point. Each point was adaptively deformed further to the 4DCT images at the end of inspiration (phase 0 CT) and expiration (phase 50 CT), respectively, in the treatment planning system using SmartAdapt® in Eclipse™ version 11.5 (Varian Medical Systems, USA). An example of adaptive deformation is shown in Fig. 2. This process first included rigid image fusion with the whole spine as the matching region of interest and then DIR of the whole liver. The distance of each point from phase 0 CT to phase 50 CT indicated the amplitude of respiration-induced motion of the segment where the point was located during the entire expiration period in free breathing. The quantitative data for the respiration-induced motion of each liver segment was then established in 3 dimensions including the LR, AP, and SI directions.

In terms of target delineation, the aim was to find an adequate expansion margin of ITV regarding tumors in different liver segments when only a regular helical CT image was available. This should not just be the range of amplitude mentioned above but needs to be more specific. Helical CT scans were taken during respiration and hence the position should be between the positions at phase 0 CT and phase 50 CT. In addition, they were scanned during respiration randomly so that the location was normally distributed. Therefore, an average amplitude over all patients for a segment from the helical CT to the 4DCT, plus 2 (specifically, 2.093 while the sample size was 20) standard deviations of all patients for a segment divided by the square root of 20 was required to cover more than 95% of each tumor. The final range should cover both ranges which were calculated from the helical CT to the phase 0 CT and the helical CT to the phase 50 CT. By these means, data on the expansion margin of gross tumor volume (GTV) or clinical target volume (CTV), which is the area that the physician decides to treat when the target is immobilized, to ITV for target delineation of each liver segment were then created.

### Verification

The stability of respiration during CT scanning had to be verified, since the helical CT and 4DCT scans were not done at the same time, and we assumed the helical CT scan was performed between phase 0 CT and phase 50 CT. Theoretically, the lung volume in the helical CT should be between phase 0 CT and phase 50 CT. Left, right, and total lung volumes were assessed in this study.

The accuracy of DIR regarding liver deformation must be verified, since the amplitude of motion was not measured with real fiducial markers but with representative points in the treatment planning system. The volume overlap index (VOI) metric was designed to quantitatively evaluate 2 sets of contours. It was defined as:

$$ \mathrm{VOI}=\frac{{\mathrm{V}}_{\mathrm{helical}\ \mathrm{CT}}\cap {\mathrm{V}}_{4\mathrm{DCT}}}{\left({\mathrm{V}}_{\mathrm{helical}\ \mathrm{CT}}+{\mathrm{V}}_{4\mathrm{DCT}}\right)/2} $$

V_helical CT_ was the deformed volume from helical CT, and V_4DCT_ was the manually-drawn volume by the physician. A value of 0 denoted no spatial overlap, and 1 indicated perfect agreement [20, 28, 29].

## Results

Table 2 shows the quantitative data on the respiration-induced motion of each liver segment during expiration. The average amplitude (mean ± standard deviation) over all segments and all patients was 0.6 ± 3.0 mm in the LR direction, 2.3 ± 2.4 mm in the AP direction, and 5.7 ± 3.4 mm in the SI direction, respectively. The liver moved to the right, posterior, and superior during expiration, on average. The excursion was greater in the SI direction compared to the LR and AP directions. In terms of individual segments, segment 7 had the largest amplitude in the SI direction at 8.6 ± 3.4 mm. Otherwise, the segments over the lateral side had greater excursion on average in the SI direction compared to the medial segments, with segments 2, 3, 6, and 7 having 6.3 ± 4.2, 5.8 ± 2.8, 6.5 ± 3.5, 8.6 ± 3.4 mm, respectively.

**Table 2:** Amplitudes of respiration-induced liver motion of each liver segment during expiration period in free breathing

Segment	Average amplitude ± SD (mm)		
	LR	AP	SI
S1	−2.0 ± 2.6	1.0 ± 1.3	5.5 ± 2.6
S2	0.3 ± 2.2	1.2 ± 3.5	6.3 ± 4.2
S3	−0.3 ± 1.9	2.4 ± 1.4	5.8 ± 2.8
S4a	−1.4 ± 3.3	1.5 ± 2.4	3.0 ± 2.6
S4b	− 1.2 ± 1.6	2.0 ± 1.9	5.3 ± 3.4
S5	− 0.2 ± 2.1	3.2 ± 2.0	5.5 ± 2.4
S6	− 0.1 ± 4.6	2.2 ± 2.3	6.5 ± 3.5
S7	− 1.4 ± 3.8	3.5 ± 2.5	8.6 ± 3.4
S8	1.0 ± 2.6	3.3 ± 2.3	5.0 ± 3.3
mean	−0.6 ± 3.0	2.3 ± 2.4	5.7 ± 3.4

Positive values denote excursion in the left, posterior, or superior directions; Negative values, right, anterior, or inferior

*Abbreviations: LR* left-right, *AP* anterior-posterior, *SI* superior-inferior, *SD* standard deviation

Table 3 shows the suggested expansion margin of ITV for target delineation of each liver segment for a regular helical CT. To cover more than 95% of each tumor, the margins in the LR, AP, and SI directions required an average over all segments and all patients of at least 1.2 mm to the left, 2.5 mm to the right, 2.5 mm to the anterior, 2.2 mm to the posterior, 5.0 mm to the superior, and 4.2 mm to the inferior, respectively. The adequate margin was not uniform concerning different segments and directions. Segment 7 demanded 7.3 mm in the SI direction, accounting for the largest margin of all segments in any direction.

**Table 3:** The suggested asymmetrical expansion margins of ITV for target delineation of each liver segment are derived from the amplitudes of DIR from helical CT to 4DCTs

Segment	Average amplitude ± SD (mm)						ITV margin (mm) (to cover more than 95% of each tumor)		
	Helical CT to phase 0 CT			Helical CT to phase 50 CT					
	LR	AP	SI	LR	AP	SI	LR	AP	SI
S1	0.8 ± 3.1	0.4 ± 1.6	− 2.5 ± 3.9	− 1.1 ± 2.2	1.4 ± 1.8	3.0 ± 3.5	− 2.2 and 2.3	− 0.4 and 2.3	−4.4 and 4.7
S2	−1.1 ± 2.5	−0.6 ± 3.7	−2.4 ± 4.9	−0.8 ± 2.6	0.6 ± 2.1	3.9 ± 4.1	− 2.3 and 0.5	−2.4 and 1.6	− 4.7 and 5.9
S3	−0.3 ± 2.9	−1.8 ± 3.1	−2.9 ± 3.5	− 0.7 ± 3.1	0.6 ± 3.0	2.9 ± 2.5	− 2.2 and 1.1	−3.3 and 2.1	− 4.6 and 4.1
S4a	0.4 ± 2.8	−1.4 ± 2.1	−0.6 ± 3.9	−1.0 ± 1.7	0.1 ± 2.4	2.4 ± 3.4	−1.8 and 1.8	−2.4 and 1.3	− 2.4 and 4.0
S4b	−0.5 ± 1.5	− 1.7 ± 2.2	−2.4 ± 3.5	− 1.8 ± 1.6	0.3 ± 2.2	2.9 ± 3.3	− 2.6 and 0.3	−2.8 and 1.4	− 4.1 and 4.5
S5	−2.2 ± 1.7	−1.4 ± 2.1	−1.9 ± 2.2	− 2.3 ± 1.3	1.7 ± 2.4	3.7 ± 2.1	−3.0 and 0.0	−2.4 and 2.9	− 3.0 and 4.7
S6	0.2 ± 4.3	− 1.2 ± 2.3	−3.2 ± 2.9	0.1 ± 4.1	1.0 ± 2.3	3.3 ± 3.7	− 1.9 and 2.3	−2.3 and 2.1	−4.6 and 5.1
S7	0.3 ± 4.2	− 1.7 ± 2.8	−3.6 ± 4.4	−1.1 ± 2.6	1.8 ± 2.1	5.0 ± 4.8	− 2.4 and 2.3	−3.1 and 2.8	−5.7 and 7.3
S8	−2.2 ± 2.5	− 1.5 ± 2.6	−1.9 ± 3.4	−1.3 ± 1.5	1.8 ± 2.5	3.2 ± 3.1	−3.4 and 0.0	−2.8 and 3.0	− 3.5 and 4.7
mean	−0.5 ± 3.1	− 1.2 ± 2.6	−2.4 ± 3.7	−1.1 ± 2.5	1.0 ± 2.4	3.4 ± 3.5	− 2.5 and 1.2	−2.5 and 2.2	− 4.2 and 5.0

*Abbreviations:* LR = left-right; AP = anterior-posterior; SI = superior-inferior; SD = standard deviation; ITV = internal target volume; CT = computed tomography

Positive values denote DIR from helical CT to 4DCT, and ITV expansion in the left, posterior, or superior directions; Negative values, right, anterior, or inferior

The mean volumes of left, right, and total lung in helical CT were 1104.6 ml, 1444.7 ml, and 2549.36 ml, respectively. All of them were between phase 0 CT (1167.0 ml, 1529.8 ml, and 2696.8 ml) and phase 50 CT (1034.3 ml, 1336.1 ml, and 2370.4 ml), showing the stability of respiration among the patients during the CT scans. The VOI of whole liver adaptive deformation was 0.94 for helical CT and phase 0 CT, and 0.96 for helical CT and phase 50 CT, which showed DIR to be quite accurate as the measurement method.

## Discussion

Liver motion caused by respiration is a serious problem during radiotherapy. In general, the strategies to reduce the impact of respiratory motion fall into three broad categories: motion-encompassing methods to accommodate the tumor motion with a large irradiation field, motion control to minimize the amplitude of the tumor motion, and motion gating or tracking to trace the tumor motion [30]. The report of American Association of Physicists in Medicine (AAPM) Task Group 76, *Managing Respiratory Motions in Radiation Oncology*, documents the details of these methods to account for respiratory motion in radiotherapy [31]. The motion-encompassing method is easy on the patient, while motion control requires cooperation of the patient, and motion gating or tracking needs sophisticated equipment and technology. However, the motion-encompassing method typically irradiates a higher volume of adjacent normal tissue. It also requires minimal motion variability as the treatment volume is often determined based on a single pre-treatment CT.

Understanding how the liver moves can improve the delivery of radiation doses to only the area of disease. Several studies have investigated the magnitude of respiratory organ motion within the past 20 years, including some that focus on the liver. It was generally acknowledged that liver tumors moved greater in the SI direction than the LR and AP directions. Magnetic resonance imaging (MRI) was used around the year 2000 to indicate a magnitude of liver motion greater than 20 mm [5, 21], which was larger than the result revealed by later studies. A real-time tumor-tracking radiotherapy system (RTRT) with the insertion of a fiducial gold marker is a novel method of treating moving tumors with a small margin and accurate dose delivery, which Japanese researchers utilized to assess the motion of liver tumors. They found tumor motion to be significantly larger in the LR and AP directions when the tumor was in the right lobe, the patient had cirrhotic liver, or the patient had no history of liver surgery [22, 23]. Case et al. reported that the change in inter- and intra-fraction liver motion was minimal during liver stereotactic body radiotherapy (SBRT), with mean liver motion amplitude of 8.0 mm in the SI direction [24]. In 4-dimensional radiotherapy, 4DCT is an essential component that allows safe reduction of the clinical target volume margin to increase the dose to the tumor and decrease the dose to normal tissue [32]. It was widely employed to evaluate organ motion for a decade, using either 4DCT or 4-dimensional cone-beam computed tomography (4DCBCT) with or without DIR or fiducial marker insertion. A Korean study showed significantly reduced liver and pancreas motion in the prone position under 4DCT estimation, especially in the SI direction [25]. While using 4DCT or 4DCBCT with fiducial marker insertion for measurement, liver motion in the SI direction was up to 17.9 mm but only 4.5 to 5.3 mm during abdominal compression. In addition, all of the markers that moved cranially also moved posteriorly and vice versa, irrespective of their location. The LR motion had a more variable relationship with the AP and SI motions [26, 27]. These results are quite compatible with our study, in which all segments moved posteriorly and superiorly during expiration but displayed diversity in the LR direction. Several studies applied 4DCT with DIR to analyze liver motion. Velec et al. deformed CT contours from exhale to inhale using Morfeus, a multi-organ biomechanical model–based DIR algorithm [15]. Hallman et al. propagated contours that were mostly defined at the phase 30 CT to all other phases using a 3-stage multi-resolution B-spline method [17]. Tai et al. populated liver contours from the 3DCT to the 4DCT with ABAS, an autosegmentation software tool based on DIR from Elekta [16]. The results of using 4DCT with DIR demonstrated liver movement from 7.9 to 10 mm in the SI direction. Table 4 summarizes the references regarding respiration-induced liver motion.

**Table 4:** Summary of respiration-induced liver motions in the references and the present study

Reference	Year	Method	Number of patient	Magnitude of motion SI direction (mm)	Abdominal compression	Individual segment evaluation
[5]	1999	MRI	1	21	No	No
[21]	2003	MRI	12	24.4	No	No
[22]	2003	RTRT w/ fiducial	20	8 (left) 9 (right)	No	No
[23]	2009	RTRT w/ fiducial	6	15.98	No	No
[24]	2010	CBCT	29	8	15/29	No
[25]	2007	4DCT	9	15 (supine) 12.5(prone)	No	No
[26]	2012	4DCT CBCT w/ fiducial	20	17.9 (4DCT) 16.5 (CBCT)	No	No
[27]	2017	4DCBCT w/ fiducial	10	5.3 (planning simulation 4DCBCT) 4.5 (pre-SBRT 4DCBCT)	10/10	No
[15]	2011	4DCT w/ DIR	21	10	6/21	No
[17]	2012	4DCT w/ DIR	18	9.7	No	No
[16]	2013	4DCT w/ DIR	15	7.9	No	No
Present study		4DCT w/ DIR	20	8.6 (Segment 7)	No	Yes

*Abbreviations: MRI* magnetic resonance imaging, *RTRT* real-time tumor-tracking radiotherapy system, *CBCT* cone-beam computed tomography, *4DCT* 4-dimensional computed tomography, *4DCBCT* 4-dimensional cone-beam computed tomography, *DIR* deformable image registration, *SBRT* stereotactic body radiotherapy, *SI* superior-inferior

A large variation in the motion was observed. While the distance between the locations increased, the difference in the absolute range of motion also increased [26]. Our study is the only one to evaluate liver motion regarding each segment and to render the individual segment expansion margin of ITV. Other than the discoveries that all segments move posteriorly and superiorly during expiration and that the largest amplitude of motion occurs in the SI direction due to physiology, we found that segments over the lateral side had greater excursion in the SI direction, which makes the liver move like a flying “seagull” during respiration. This is probably due to physiology and anatomy as well as the lateral segments having less anatomical fixation, such as being attached to a ligament or great vessels. Segment 7 is the lateral segment at the liver dome and thus has the greatest amplitude. Regarding ITV expansion, although the coverage rate by the margins is only approximately 95%, this is good enough in our daily practice. Time, facility, and personnel costs can also be saved by expanding contours using the data.

To analyze liver motion by means of 4DCT, the ITV including all phases being analyzed is the most accurate. However, delineating two extreme phases at end-inspiration and end-expiration is a reasonably safe and labor-saving method of deriving the ITV. Xi et al. compared ITV values derived from the contouring of 2 phases (0%, 50%), 3 phases (0%, 20%, 50%), 4 phases (0%, 20%, 50%, 80%), and 6 phases (0%, 10%, 20%, 30%, 40%, 50%) to all 10 phases of 4DCT. All of the values showed excellent agreement, with encompassing ratios of 94.1 ± 1.8%, 95.2 ± 1.5%, 96.5 ± 1.5%, and 97.6 ± 0.7%, respectively. The 3D shift between the centers of mass of 2-phase and 10-phase 4DCT was only 0.6 mm. Otherwise, 2-phase 4DCT had very good tumor coverage in the SI direction, and the surface distance for voxels missing was only 1.7 ± 0.8 mm in the LR and AP directions [33].

Although the majority of DIR algorithms perform at an accuracy equivalent to the voxel size and are promised to improve treatment planning, delivery, and assessment, there are discrepancies in different shifts. The ranges in average absolute error for liver 4DCT were 0.8–1.5 mm (LR), 1.0–5.2 mm (AP), and 1.0–5.9 mm (SI), respectively, in a multi-institution deformable registration accuracy study [34]. To quantify the accuracy of individual DIR algorithms, Velocity AI—a commercially available DIR tool—was analyzed and shown to decrease the extent of observable misalignments to 1–4 mm in general. The DIR tool is capable of reducing a mean target registration error to a clinically acceptable level [35]. Kirby et al. used an automated deformable image registration evaluation of confidence tool (AUTODIRECT)—a software tool used to evaluate DIR accuracy—to predict DIR errors [36]. Kim et al. tested the AUTODIRECT framework, which showed promise for estimating DIR-driven dose-mapping errors [37].

To validate the accuracy and usefulness of automatic contour propagation using SmartAdapt®, most studies have applied VOI as the quantification method. Ramadaan et al. assessed SmartAdapt® in the context of head and neck CT registration and achieved overall VOI results of 0.82 ± 0.08, concluding that propagated structures were acceptable for clinical settings [20]. Thor et al. examined SmartAdapt® in the context of pelvic CT registration, yielding VOI values of 0.80 for the prostate, 0.77 for the rectum, and 0.73 for the bladder [38]. Konig et al. integrated a local rigidity deformation model into a DIR algorithm, which showed VOI values of 0.79 for the prostate and 0.86 for the bladder [29]. Our study demonstrated VOI values of 0.94 for helical/phase 0 CT and 0.96 for helical/phase 50 CT in the whole liver contour propagations. Although not directly indicating the adaptive deformation of representative points, the excellence of the VOI values found expresses the reliability of applying DIR to liver CT.

This study analyzed 20 eligible patients. Theoretically, when more patients are analyzed, the average amplitude will not change but the standard deviation will be smaller. Therefore, the individual segment expansion margin of ITV to cover more than 95% of each tumor, which is calculated by the formula in the methodology, will also be smaller due to the larger sample size and the smaller standard deviation. That means that the larger the sample size is, the more confined the ITV expansion margin that could be found. The margins we rendered are based on the 20 patients investigated in the study. Although the margins will be theoretically smaller if concerning the entire liver population, for treatment safety, the margins are not recommended to be narrowed down for clinical application before more patients are analyzed. One limitation of this study is that only the population variability can be taken into account with the methodology, but not the intrafraction or interfraction variability, which can be significant, especially for lesions moving with amplitudes above 7 mm [39]. Another limitation is hysteresis, that is, differences in the inhale-to-exhale and exhale-to-inhale tumor trajectory. As only end-inspiration and end-expiration were analyzed, this additional motion should also be considered. The margins suggested in the manuscript are for GTV or CTV to ITV expansion. However, the margins for ITV to planning target volume (PTV) expansion, regarding other set-up errors, have not been taken into account. Concerning all of them together, it is feasible to add an extra margin to the suggested expansion margin of ITV to final PTV in clinical application.

## Conclusion

Respiration-induced liver motion varies widely with respect to different liver segments. The greatest excursion occurred in segment 7, followed by the segments over the lateral side in the SI direction. An individual segment expansion margin of ITV is required for target delineation regarding each segment and direction to cover the range of respiration-induced motion during radiotherapy.

## Abbreviations

4DCT: 4-Dimensional computed tomography
AAPM: American association of Physicists in Medicine
AP: Anterior-Posterior
CT: Computed tomography
DIR: Deformable image registration
DLBCL: Diffuse Large B-Cell Lymphoma
ITV: Internal target volume
LR: Left-Right
MRI: Magnetic resonance imaging
NTCP: Normal tissue complication probability
RILD: Radiation-Induced Liver Disease
RPM: Real-time position management
RTRT: Real-time Tumor-tracking Radiotherapy system
SI: Superior-Inferior
VOI: Volume overlap index
